# Implementing fast-track in total hip arthroplasty: rapid mobilization with low need for pain medication and low pain values: Retrospective analysis of 102 consecutive patients

**DOI:** 10.1007/s00393-021-00978-5

**Published:** 2021-03-11

**Authors:** Julia Sabrina Götz, Franziska Leiss, Günther Maderbacher, Matthias Meyer, Jan Reinhard, Florian Zeman, Joachim Grifka, Felix Greimel

**Affiliations:** 1 Department of Orthopedics, University Medical Center Regensburg, Asklepios Klinikum Bad Abbach, Kaiser-Karl-V.-Allee 3, 93077 Bad Abbach, Germany; 2 Center for Clinical Studies, University Medical Center Regensburg, Regensburg, Germany

**Keywords:** Pain management, Total hip arthroplasty, Range of motion, Fast-frack, Enhanced recovery, Schmerz-Management, Hüftgelenktotalendoprothetik, Beweglichkeit, Fast-Track, Rasche Rekonvaleszenz

## Abstract

**Introduction:**

Total hip arthroplasty (THA) is reported to be one of the most painful surgical procedures. Perioperative management and rehabilitation patterns are of great importance for the success of the procedure. The aim of this cohort study was the evaluation of function, mobilization and pain scores during the inpatient stay (6 days postoperatively) and 4 weeks after fast-track THA.

**Materials and methods:**

A total of 102 consecutive patients were included in this retrospective cohort trial after minimally invasive cementless total hip arthroplasty under spinal anesthesia in a fast-track setup. The extent of mobilization under full-weight-bearing with crutches (walking distance in meters and necessity of nurse aid) and pain values using a numerical rating scale (NRS) were measured. Function was evaluated measuring the range of motion (ROM) and the ability of sitting on a chair, walking and personal hygiene. Furthermore, circumferences of thighs were measured to evaluate the extent of postoperative swelling. The widespread Harris Hip Score (HHS) was used to compare results pre- and 4 weeks postoperatively.

**Results:**

Evaluation of pain scores in the postoperative course showed a constant decrease in the first postoperative week (days 1–6 postoperatively). The pain scores before surgery were significantly higher than surgery (day 6), during mobilization (*p* < 0.001), at rest (*p* < 0.001) and at night (*p* < 0.001). All patients were able to mobilize on the day of surgery. In addition, there was a significant improvement in independent activities within the first 6 days postoperatively: sitting on a chair (*p* < 0.001), walking (*p* < 0.001) and personal hygiene (*p* < 0.001). There was no significant difference between the measured preoperative and postoperative (day 6 after surgery) thigh circumferences above the knee joint. Compared to preoperatively, there was a significant (*p* < 0.001) improvement of the HHS 4 weeks after surgery. In 100% of the cases, the operation was reported to be successful and all of the treated patients would choose a fast-track setup again.

**Conclusion:**

Application of a fast-track scheme is effective regarding function and mobilization of patients. Low pain values and rapid improvement of walking distance confirms the success of the fast-track concept in the immediate postoperative course. Future prospective studies have to confirm the results comparing a conventional and a fast-track pathway.

## Abstract (de)

**Einführung:**

Die Hüftgelenktotalendoprothetik zählt zu den schmerzhaften chirurgischen Eingriffen, und perioperative Management- und Rehabilitationsvorgehensweisen sind für den Erfolg von großer Bedeutung. Ziel dieser Studie war die Evaluierung von Funktion, Mobilisierung und Schmerzwerten während des stationären Aufenthalts (6 Tage postoperativ) und 4 Wochen nach Fast-Track-Hüftgelenkendoprothetik.

**Material und Methoden:**

In diese retrospektive Studie wurden 102 Patienten nach minimal-invasiver zementfreier Hüftendoprothesenimplantation unter Spinalanästhesie in einem Fast-Track-Setup eingeschlossen. Der Umfang der Mobilisierbarkeit bei Vollbelastung mit Krücken (Gehstrecke [m] und Pflegebedürftigkeit) sowie Schmerzwerte wurden unter Verwendung einer numerischen Bewertungsskala (NRS) gemessen. Die Funktion wurde bewertet, indem die Beweglichkeit (ROM), die Fähigkeit zum Sitzen auf einem Stuhl und zum Gehen sowie die Durchführung persönlicher Hygienemaßnahmen analysiert wurden. Darüber hinaus wurden die Oberschenkelumfänge gemessen, um das Ausmaß der postoperativen Schwellung zu objektivieren. Der weit verbreitete Harris Hip Score (HHS) wurde verwendet, um die Ergebnisse vor sowie 4 Wochen nach der Operation zu vergleichen.

**Ergebnisse:**

Die Auswertung der Schmerzwerte im postoperativen Verlauf zeigte eine konstante Abnahme in der ersten postoperativen Woche (Tage 1–6 postoperativ). Die Schmerzwerte vor der Operation waren signifikant höher als nach der Operation (Tag 6), während der Mobilisierung (*p* < 0,001), sowie in Ruhe (*p* < 0,001) und in der Nacht (*p* < 0,001). Alle Patienten konnten am Tag der Operation mobilisiert werden. Zusätzlich gab es innerhalb der ersten 6 Tage nach der Operation eine signifikante Verbesserung bei Sitzen auf einem Stuhl (*p* < 0,001), Gehen (*p* < 0,001) und Durchführung persönlicher Hygienemaßnahmen (*p* < 0,001). Es bestand kein signifikanter Unterschied zwischen dem präoperativ und postoperativ gemessenen Oberschenkelumfang oberhalb des Kniegelenks (Tag 6 nach der Operation). Im Vergleich mit dem präoperativen Score zeigte sich 4 Wochen nach der Operation eine signifikante (*p* < 0,001) Verbesserung des HHS. Alle Patienten gaben an, dass die Operation erfolgreich gewesen sei und dass sie sich wieder für die Fast-Track-Vorgehensweise entscheiden würden.

**Fazit:**

Ein Fast-Track-Schema ist hinsichtlich Funktion und Mobilisierung von Patienten erfolgreich umzusetzen. Niedrige Schmerzwerte und eine schnelle Verbesserung der Gehstrecke bestätigen den Erfolg des Fast-Track-Konzepts im unmittelbaren postoperativen Verlauf. Künftige prospektive Studien müssen die Ergebnisse bestätigen, nach Möglichkeit im Vergleich eines konventionellen und eines Fast-Track-Vorgehens.

## Introduction

Fast-track total hip arthroplasty (THA) is a well-established concept including optimized logistics and evidence-based treatment options. Focusing on minimizing surgical stress and improved postoperative recovery leads to lower mortality and morbidity as well as high patient satisfaction [1]. In general, all patients are potentially eligible for fast-track THA. Hence, fast-track concepts have evolved and are standard in numerous joint replacement facilities. This has been achieved by a well-defined multidisciplinary setup, which has to be carefully attended by every medical department involved. Generally speaking, fast-track THA enables an accelerated perioperative course [2].

In the USA and most European countries more than 200 total hip arthroplasties are implanted per 100,000 inhabitants every year. Numbers of up to 300/100,000 were reported (in Germany) in November 2019, published by the Organization for Economic Co-operation and Development (OECD) [3]. The key factor for patient’s satisfaction is pain, as measured using the visual or numerical scale. Total hip arthroplasty (THA) is ranked 11th with respect to the most painful surgical procedures and it is also a stressful surgical intervention [4–6]. Less pain is associated with early mobilization; furthermore, it may lower costs because of a shorter hospital stay and less burden on the health care system [7]. Major surgery, including total hip arthroplasty (THA), is followed by a convalescence period, during which the loss of muscle strength and function is considerable, especially early after surgery [8, 9]. Therefore, early rehabilitation is important [10]. The pathophysiologic and clinical consequences of early rehabilitation interventions include less catabolism and loss of postoperative muscle mass and function, improved pulmonary function, enhanced recovery of gastrointestinal function and reduced thromboembolic complications [11, 12]. Recent data indicate that physiotherapy exercise, including strength training, can be initiated early (earlier) after THA [13, 14]. Multidisciplinary cooperation is therefore of great importance.

Furthermore, patients express concern about pain and dependence upon other persons during the postoperative and rehabilitation period after the surgical procedure [5]. Therefore, it is important to know and understand the timing of postoperative pain and “aid-dependence” in order to also improve patients’ education preoperatively. The key issues of fast-track programs are patient motivation and transfer of partial responsibility to the patient through intensive information. An optimized surgical technique and modern multimodal pain therapy enable early mobilization [15].

The aim of this study was to evaluate the incidence and intensity of postoperative pain, the mobility and the function at the early and 4‑week follow-up after fast-track THA.

## Materials and methods

In the present study, 102 consecutive patients were retrospectively evaluated. Inclusion criteria were all patients after primary cementless THA with spinal anesthesia within a newly established fast-track pathway. Exclusion criteria were the existence of chronic pain syndrome preoperatively, except the reported pain of the hip joint arthritis. Furthermore, intraoperative change to general anesthesia was an exclusion criterion in order to prevent possible bias by the anesthetic method used. The interventions were performed between mid 2018 and mid 2019 after a fast-track implementation phase in early 2018. The fast-track scheme was initially performed parallel to a conventional pathway in order to enable a smooth transition.

General data (age and sex) were recorded as well as the ASA risk classification (American Society of Anesthesiologists). The fast-track scheme was performed as follows: preoperative multidisciplinary lecture and gait-training with crutches for all patients, pre-emptive NSAID (Non-Steroidal Anti-Inflammatory Drugs) administration (etoricoxibe 90 mg once one hour before the intervention), minimally invasive anterolateral approach under spinal anesthesia (prilocaine 1% hyperbaric 4 ml = 80 mg and sufentanil 10 µg as standard), i.v. application of dexamethasone (8 mg), implantation of cementless hip arthroplasty components (cementless DePuy Pinnacle® cup, cementless DePuy Corail® stem, DePuy Inc., Warsaw, IN, USA), use of local infiltration analgesia in the periacetabular and -femoral region as well as subcutaneous infiltration (200 mg ropivacaine, for deep infiltrations with 0.5 mg adrenalin), administration of tranexamic acid (1 g intravenously and 2 g topically), renunciation of suction drains, conventional wound closure stitching plus application of wound glue. A transparent wound dressing was applied. Full weight bearing was allowed and range of motion was not restricted. Mobilization started as soon as peripheral sensory and motoric skills were approved, usually 1–2 h after surgery. After cardiovascular stimulating and thrombosis prophylaxis exercises, first walking exercises with crutches were started under physiotherapeutic supervision. Target for the day of surgery was a walking distance of at least 50 m. Starting on postoperative day 1, a fast-track exercise circuit was used to extend exercise intensity; patients received additional physiotherapy twice a day. Physiotherapy included mobilization of the hip, muscle strengthening as well as thrombosis and pneumonia prophylaxis. The fast-track exercise circuit consisted of a walking course, various muscle strengthening exercises and tutorials to improve coordination. In addition, a mirror wall with a holding bar was used at the ward. Here, the patients were able to repeat the exercises several times a day, independently and with self-control, in order to reflect their gait pattern and correct possible errors themselves.

A standardized pain management concept was applied regarding the WHO recommendations [16]. At the recovery unit immediately postoperatively, nurses administered 3 mg of piritramide if necessary, depending on numerical rating scale (NRS; 0 = no pain; 10 = worst pain imaginable) values. For oral controlled analgesia, ibuprofen (600 mg) was administered 3 times daily and metamizole (500 mg) 4 times daily on a regular basis. Depending on NRS values, patients received tramadol 100 mg (40 drops) and oxycodone 10 mg as optional additional analgetic medication, if necessary. In cases of persisting pain, the nurse had to notify the physician for further advice. Pain scores were evaluated preoperatively, separated into pain while resting, moving and at night, using the NRS. A mean pain score was calculated per day for graphic representation.

In Germany, the direct discharge to a rehabilitation unit is usually planned after the 6th postoperative day. Upon discharge, patients had to be able to walk 100 m safely under full weight bearing, take stairs and take care of themselves independently.

The patient’s independent activities per day in minutes (sit on a chair, go for a walk and personal hygiene), range of motion (in degrees) and the leg circumference (in centimeters) were evaluated preoperatively, on the day of surgery and from days one to six after the operation by specially trained physiotherapists. Range of motion was measured as passive and active range of motion: extension, flexion and abduction. A mean of active and passive range of motion was calculated per day for graphic representation. Furthermore, the maximum range of motion during the day was documented. The thigh circumference was measured 15 cm, 20 cm and 30 cm above the knee joint to objectify swelling, with regard to the method of the German employer’s liability insurance association. All these parameters allowed the study group to evaluate detailed information of mobilization and pain values in the immediate postoperative course during the first postoperative week.

In addition, the Harris Hip Score (HHS) [17] was assessed before and 4 weeks after surgery to evaluate overall clinical performance. The score contains ten items covering four domains. The domains are pain, function, absence of deformity, and range of motion. The higher the score, the better is the outcome for the individual. The maximum possible score is 100. Results can be interpreted with the following: < 70 = poor result; 70–80 = fair, 80–90 = good, and 90–100 = excellent. At hospital discharge, the patients had to evaluate whether they rate the operation successful (“Yes” or “No”), if they would choose fast-track procedure again (“Yes” or “No”) and how the patients rate their overall well-being (much better = 1; better = 2; equal = 3; worse = 4 and much worse = 5).

The study was approved by the local ethics committee (IRB approval number 19-1352-104). The study was applied in accordance with the ethical standards of the 1975 Declaration of Helsinki.

Statistical analysis was performed by using Excel (version 16, Microsoft, Redmond, WA, USA). For descriptive analysis, absolute and relative frequencies or mean and standard deviation were stated. A t-test for paired samples was used for statistical evaluation. No imputation methods were used. A *p*-value < 0.05 was considered statistically significant.

## Results

The cohort of 102 patients composed of 68 men (66.0%) and 35 women (34.0%) with an average age of 61.3 ± 10.9 years (60.4 ± 10.5 for men, 62.8 ± 11.7 for women). None of the included 102 patients had a second surgical intervention due to a complication. The average duration of surgery was 63.6 (±11.9) min with a minimum of 44 min and a maximum of 110 min. Furthermore, 28.4% had an ASA score of one, 64.7% an ASA score of two and 6.9% an ASA score of three; none of the patients had a higher ASA score. The average ASA score was 1.8 (±0.6).

### Pain

**Fig. 1:**
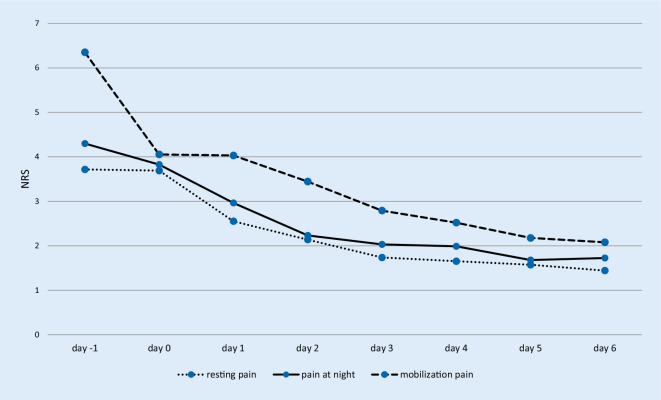
Mean values of resting pain, pain at night and movement pain: Mean values *n* = 102. The x‑axis represents the days (Pre-surgery [Pre-OP], day of surgery [OP-Day] and day one to six after the surgery). The y‑axis represents the numerical rating scale (NRS)

Fig. 1 and Table 1 illustrate pain values preoperatively, on the day of the surgical procedure and days one to six after the operation. There was a highly significant difference between the pain before the operation and the 6th day after the operation during mobilization, as well as at rest and at night (*p* < 0.001, respectively).

**Table 1:** Resting pain, pain at night and mobilization: Mean values (standard deviation, maximum and minimum pain), (*n* = 102)

Subjective pain interpretation			
	Rest	Night	Mobilization
Day −1	3.7 (±2.4; 0–10)	4.3 (±2.5; 0–10)	6.4 (±2.1; 0–10)
Day 0	3.7 (±2.4; 0–9)	3.8 (±2.3; 0–9)	4.1 (±2.3; 0–9)
Day 1	2.6 (±2.0; 0–8)	3.0 (±2.2; 0–9)	4.0 (±2.1; 0–9)
Day 2	2.1 (±2.0; 0–9)	2.2 (±2.0; 0–8.5)	3.4 (±1.9; 0–7)
Day 3	1.7 (±1.7; 0–7)	2.0(±2.0; 0–8.5)	2.8 (±1.8; 0–8)
Day 4	1.7 (±1.8; 0–8.5)	2.0 (±1.9; 0–8.5)	2.5 (+±1.8; 0–9)
Day 5	1.6 (±1.7; 0–6.5)	1.7 (±1.9; 0–7)	2.2 (±1.7; 0–6)
Day 6	1.4 (±1.6; 0–6)	1.7 (±1.9; 0–7)	2.1 (±1.6; 0–6)

There was no significant difference regarding pain values between men and women preoperatively (*p* = 0.5) and 6 days postoperatively (*p* = 0.5): Mean pain value for men was 6.3 ± 2.0 preoperatively and for women 6.4 ± 2.2, while it decreased to a mean pain value 6 days postoperatively for men (2 ± 1.6) and for women (2 ± 1.5).

### Range of motion

**Fig. 2:**
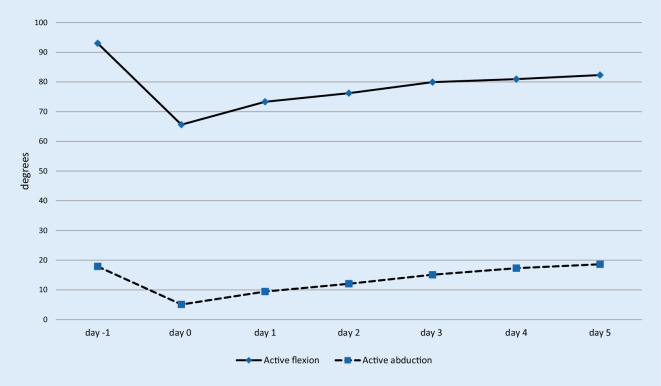
Mean values active range of motion: *n* = 102. The x‑axis represents the days (Pre-surgery [Pre-OP], and day one to six after the surgery). The y‑axis shows the range of motion in degrees, measured by the physiotherapists

Fig. 2 and Table 2 show the range of motion preoperatively and from day one to six after the operation. On day six after the operation, it was observed that patients have a better passive flexion and abduction mobility than preoperatively. There was a highly significant improvement comparing the range of motion before the surgery and on the 6th day after the operation: passive flexion (*p* < 0.001) and passive abduction (*p* < 0.001). Active flexion (*p* = 0.9) and active abduction (*p* = 0.5) showed no significant difference.

**Table 2:** Active range of motion: Mean values in degrees (standard deviation, maximum and minimum),* n* = 102

	Active flexion	Active abduction
Day −1	93.0 (±15.6; 50–125)	17.9 (±7.6; 0–40)
Day 1	65.6 (±17.3; 0–80)	5.1 (±8.2; 0–30)
Day 2	73.3 (±12.6;0–85)	9.5 (±8.8; 0–30)
Day 3	76.2 (±10.1;0–90)	12.1 (±9.0; 0–30)
Day 4	79.9 (±6.1;60–90)	15.1 (±9.3; 0–30)
Day 5	81.0 (±7.1;45–90)	17.3 (±9.8; 0–40)
Day 6	82.3 (±4.8;70–90)	18.6 (±9.0; 0–40)

### Thigh circumference

**Fig. 3:**
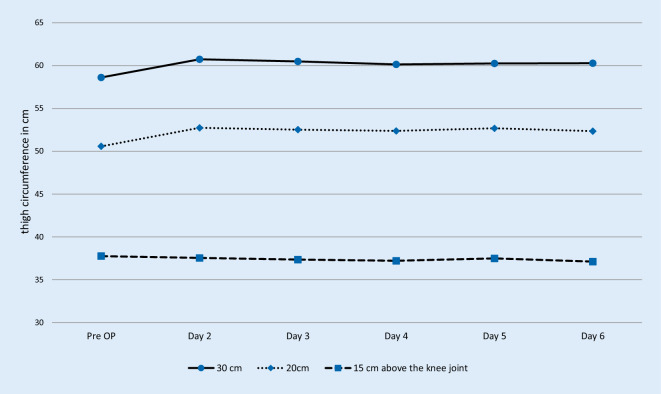
Thigh circumference 30 cm, 20 cm and 15 cm above the knee joint: Mean values, *n* = 102. The x‑axis represents the days (Pre-surgery [Pre-OP] and day two to six after surgery). The y‑axis represents the circumference in cm

Fig. 3 and Table 3 show the thigh circumference preoperatively and from days two to six after the operation, measured in centimeters. A mean was calculated per day for graphic representation. There was no significant difference between the preoperative and postoperative (day 6 after surgery) thigh circumference 15 cm (*p* = 0.34), 20 cm (*p* = 0.05) and 30 cm (*p* = 0.09) above the knee joint.

**Table 3:** Thigh circumference 30 cm, 20 cm and 15 cm above the knee joint in cm: Mean values (standard deviation, maximum and minimum), *n* = 102

Thigh circumference above the knee joint			
	30 cm	20 cm	15 cm
Day −1	58.6 (±6.3; 42–78)	50.6 (±5.8; 30–68)	37.8 (±4.1; 25–48)
Day 2	60.7 (±6.3; 45–81)	52.7 (±6.4; 38–81)	37.5 (±3.8; 29–47)
Day 3	60.5 (±6.1; 45–77)	52.5 (±6.0; 37–69)	37.3 (±4.0; 29.5–47)
Day 4	60.2 (±6.1; 45–79)	52.4 (±5.6; 38–69)	37.2 (±3.9; 28–47)
Day 5	60.3 (±6.3; 46–78)	52.7 (±5.6; 40–68)	37.5 (±4.3; 28–48)
Day 6	60.3 (±6.3; 46–78)	52.4 (±5.7; 40–68)	37.1 (±4.5; 26–48)

## Activities

**Fig. 4:**
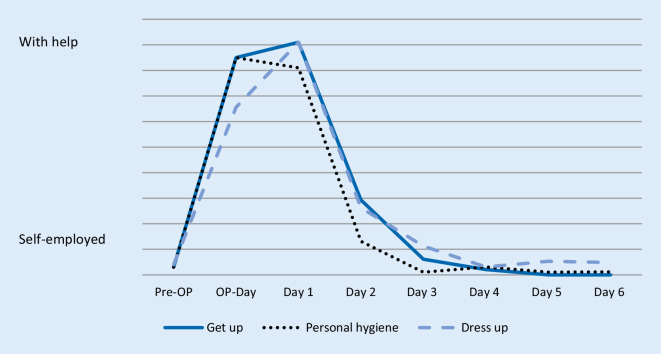
Patient independence, mean values, *n* = 102. The x‑axis represents the days (Pre-surgery [Pre-OP], day of surgery [OP-Day] and day one to six after surgery). The y‑axis represents patient independence. The patients can classify their subjectively independence (get up, personal hygiene and dress up) from “self-employed” to “with help”

**Fig. 5:**
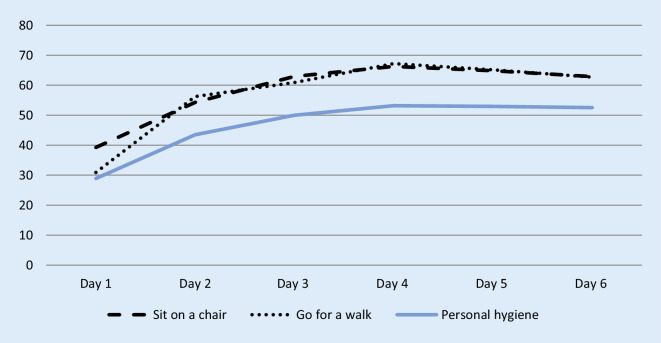
Patient independent activities per day in minutes: Mean values, *n* = 102. The x‑axis represents the days (Day one to six after surgery). The y‑axis represents the patient independent activities (sit on a chair, go for a walk and personal hygiene) in minutes

Fig. 4 shows patient reported independence (get up, personal hygiene and dress) from “self-employed” to “with help”. Values were not significantly different. Finally, Fig. 5 demonstrates patients’ independent activities (sit on a chair, go for a walk and personal hygiene) per day in minutes. There was a significant improvement in independent activities within the first 6 days postoperatively: sit on a chair, go for a walk and personal hygiene (*p* < 0.001, respectively). In Figs. 4 and 5 it can be seen that study participants were able to carry out personal hygiene independently, with an average of 43.5 min, from the 2nd postoperative day.

### Outcome

There was a significant (*p* < 0.001) improvement in the Harris Hip Score (HHS) comparing preoperatively and four weeks after surgery (Table 4). In 100% of the cases, the operation was described to be successful and all of the operated patients would choose the same fast-track procedure again. On average, patients felt “much better” (mean value = 1, ±1) on hospital discharge, compared to preoperatively.

**Table 4:** The Harris Hip Score (HHS). The maximum possible score is 100. Results can be interpreted as following: < 70 = poor result; 70–80 = fair, 80–90 = good, and 90–100 = excellent

Harris Hip Score (HHS)				
	Mean value	Standard deviation	Maximum	Minimum
Before surgery	54.0	±13.7	91	27
After surgery (4 weeks)	74.4	±13.1	97	38

## Discussion

In the study at hand, more men (66.0%) than women (34.0%) underwent total hip arthroplasty (THA). This observation correlates with the results of the 10 included studies (9936 cases) in the systematic review of Zhu et al. [18]. Our study results demonstrate no significant difference concerning NRS pain levels [10] between men and women preoperatively (*p* = 0.5) and 6 days postoperatively (*p* = 0.5). This finding is contrary to the results by Wiesenfeld-Hallin. Wiesenfeld-Hallin postulates that the male sex is less sensitive to pain than the female [19]. Numerous studies described the evidence of a gender-related relationship with postoperative pain [20–23]. Other causes are given by psychological, biological and sociocultural factors. Furthermore, a higher rate and severity of metal sensitization are reported with more pain in women after arthroplasty. According to Caicedo et al. [24], the cause might be the immunological preset.

A major risk factor for the occurrence of joint disease is age in general. The prevalence of hip arthritis increases with age; in the 7th life decade it reaches a plateau [25]. The average age of the present patient population of 61.3 ± 10.9 years is congruent with the results of the study by Schrader et al. [26]. They found that 67.3% of THA were implanted in patients aged 60–79 years.

There are numerous studies demonstrating a highly significant decrease in NRS from preoperatively to 6 to 12 months after THA [27, 28]. Within the first 3 postoperative months most of the pain improvements evolve [16, 25–29]. Lenguerrand et al. [27] described a highly significant pain decrease in the first 3 months postoperatively. Greimel et al. examined the course of pain after total hip arthroplasty within a standardized pain management concept: “Evaluation of pain scores in the postoperative course showed a constant decrease in the first postoperative week (mean NRS 3.1 on day 1 to mean NRS 2.3 on day 8) and, then, a perpetual increase for 3 days (mean NRS 2.6 on day 9 to mean NRS 2.3 on day 12)” [16]. Regarding the course of postoperative pain in the first six days (mean NRS 3.7 on day of surgery to mean NRS 1.4 on day 6) after fast-track THA, a significant and continuous postoperative pain level decrease has been reported. In our experience, the early postoperative period is particularly challenging for the patients for socioprofessional reintegration. Our results on pain improvements after fast-track THA are consistent with previous studies [29–34]. Therefore, efficient pain therapy is necessary in this period including adaptation of medication to patient-reported pain levels.

Bandholm et al. [10] attribute a large role to physiotherapy when using a fast-track concept after THA and/or TKA. Muscle strength and functional performance in particular benefit from early physiotherapy after the surgery. This is confirmed by our results regarding motion of the operated joint. There is a significant improvement between the range of motion before surgery and the 6th day after surgery considering passive flexion (*p* < 0.001) and passive abduction (*p* < 0.001). Temporiti et al. [35] concluded that early mobilization on the day of surgery result in additional benefits in patients’ independence in the first week after THA without pain aggravation or adverse effects on hip function and quality of life. Our patients also tolerated the complete fast-track program including intensive physiotherapy. In addition, 100% of the patients would choose the same fast-track procedure again. Within one week postoperatively, the range of motion of the hip and gait performance had improved.

Klapwijk et al. [36] showed that after 6 weeks, 91% of all patients reported better function and less pain than preoperatively. After fast-track THA, these results could be achieved after just 6 days. The patients within the study at hand improved early functional outcome with significant improvement in independent activities within the first 6 days postoperatively: sit on a chair (*p* < 0.001), go for a walk (*p* < 0.001) and personal hygiene (*p* < 0.001). On average, German men need 24.6 min and women 28.1 min in the bathroom [37]. In addition, they are at least 20 min a day on the toilet [38]. This results an average hygiene time of 50 min per day. Due to the early mobilization, the study participants were able to pursue their personal hygiene routine independently on the 2nd day with an average of 43.5 min. On the 3rd day, the average of 50 min was already reached.

Bandholm et al. [10] describes a considerable reduction of knee-extension strength after fast-track THA. They found no correlation between the change of thigh or knee circumferences and the early strength reduction. An enlarged thigh circumference would indicate a hematoma, whereas a reduction of the thigh circumference would indicate muscle loss. We pointed out that there was no significant difference between the preoperative and postoperative (day 6 after surgery) thigh circumferences 15 cm (*p* = 0.34), 20 cm (*p* = 0.05) and 30 cm (*p* = 0.09) above the knee joint. The fast-track setup without using wound drains with application of tranexamic acid and under early mobilization apparently did not lead to swelling or relevant hematoma. Unfortunately, no study evaluating pre- and postoperative thigh circumferences was found whose results could have been compared to ours. However, the measuring technique is widely spread.

We demonstrated that patients can get up, get dressed and do personal hygiene completely independently on the 2nd postoperative day. On the one hand, this relieves the burden on the nursing staff, and an earlier discharge time is enabled. Larsen et al. [39] defined the fast-track protocol with mobilization and exercise started on the day of surgery, intensive mobilization of patients after presetting daily goals. Review articles such as the “Economic analyzes of fast-track total hip and knee arthroplasty” by Büttner et al. [40] show that using a fast-track protocol can significantly reduce the overall costs of treatment.

Patient-reported outcome after fast-track hip arthroplasty measured with the Harris Hip Score (HHS) showed significant improvement (54.0 vs 74.4) comparing preoperatively and four weeks after surgery. Fast-track surgery (56.20 vs 95.36) combined with a clinical nursing pathway can significantly improve the patient-reported and functional outcome of patients undergoing THA (58.09 vs 89.39) measured with Harris Hip Score before surgery and 3 weeks after [41]. In addition, Maempel et al. found significant improvement in Harris Hip Score (42.8 vs 41.5) at 12–18 months postoperatively but there was no significant difference between fast-track program and conventional THA [42].

Limitations to be mentioned are the retrospective study design, with its restriction on explanatory power. Furthermore, possible selection bias within the selected cases is possible because fast-track procedures were only performed if the individual surgeon was convinced that it would succeed—as the fast-track concept was recently established. Possibly, patients with severe comorbidities were not included in the study collective, and possibly, especially these patients may have a good benefit with regards to this specific procedure. Furthermore, reported pain course is restricted to 6 days postoperatively. Possible external influence factors on pain sensation in the postoperative period, e.g. family, daily routine and work, could therefore not be considered. Still, the standardized setup within this early postoperative period enables us to have bias-reduced insight into pain course which is clearly a strength of the study.

In addition, results in our study are limited to pain evaluation, range of motion, thigh circumference above the knee joint, activities and the HHS preoperative and postoperative after total hip arthroplasty in fast-track scheme. Long-term results such as behavior in everyday life, work and sports would also be interesting. Randomized controlled group analysis to compare fast track with a conventional rehabilitation pathway should be performed in the future to point out dedicated differences; to date and to our knowledge, none has been published.

However, the evaluation with a comparison group was not the target of this work. In the specific fast-track setting as described in this study, patients can expect early postoperative mobilization with good function, early drop of pain levels and regain of independent activities without relevant swelling. This information can be either used to improve patient education preoperatively and/or to improve the pain protocol to prevent an increase of NRS.

## Conclusion

The present single center study demonstrates pain course, range of motion, activities and circumference of the thighs to objectify swelling after primary cementless hip arthroplasty in a fast-track setup: preoperatively and within the first 6 days postoperatively. The pain scores after the operation (day 6) were significantly lower than preoperatively. Pain levels decreased steadily on the days after surgery during the hospital stay—even on the day of surgery, pain levels were lower than preoperatively. All patients were able to mobilize on the day of surgery. There was also a significant improvement in independent activities within the first 6 days postoperatively: sitting on a chair, walking and personal hygiene (*p* < 0.001, respectively). Most patients achieved their independence on day 2 after surgery. There was no significant difference between the preoperative and postoperative (day 6 after surgery) thigh circumferences 15 cm (*p* = 0.34), 20 cm (*p* = 0.05) and 30 cm (*p* = 0.09) above the knee joint; therefore no relevant postoperative swelling could be stated. There was a significant (*p* < 0.001) improvement of the HHS four weeks after surgery compared to preoperatively, meaning a “good result” with regard to the HHS value interpretation. In 100% of the cases, the operation was reported to be successful and all of the treated patients would choose a fast-track setup again, underlining the success when using a fast-track setup. Orthopedic surgeons have to take these findings into account when planning to establish a fast-track scheme. The occurrence of pain values and rapid improvement of range of motion and independent activities should be clearly explained to patients. Future prospective studies have to confirm the results, ideally with comparison of a conventional and a fast-track pathway.

## Declarations

All procedures performed in studies involving human participants or on human tissue were in accordance with the ethical standards of the institutional and/or national research committee and with the 1975 Helsinki declaration and its later amendments or comparable ethical standards. The study was approved by the local Ethics Committee with the approval number 19-1352-104 (IRB approval).
